# GAL-201 as a Promising Amyloid-β-Targeting Small-Molecule Approach for Alzheimer’s Disease Treatment: Consistent Effects on Synaptic Plasticity, Behavior and Neuroinflammation

**DOI:** 10.3390/ijms26094167

**Published:** 2025-04-28

**Authors:** Katrin Riemann, Jeldrik von Ahsen, Tamara Böhm, Martin Schlegel, Matthias Kreuzer, Thomas Fenzl, Hermann Russ, Christopher G. Parsons, Gerhard Rammes

**Affiliations:** 1 Galimedix Therapeutics Inc., 3704 Calvend Lane, Kensington, MD 20895, USA; 2 Department of Anesthesiology and Intensive Care, School of Medicine and Health, Technical University of Munich, Ismaninger Str. 22, 81675 Munich, Germany

**Keywords:** small molecule, neuroprotection, tgArcSwe mice, activation of microglia and astrocytes, neuroinflammation, toxic oligomers, synaptic plasticity, amyloid-β-induced spine loss, Aβ(p3-42), Aβ_1–40_, 3NTyr(10)-Aβ), off-pathway aggregation, prion-like mechanism, amyloid-β-aggregation-inhibitor, modulation of amyloid-β-aggregation, Alzheimer’s disease, neurodegenerative diseases

## Abstract

Soluble oligomeric forms of Amyloid-β (Aβ) are considered the major toxic species leading to the neurodegeneration underlying Alzheimer’s disease (AD). Therefore, drugs that prevent oligomer formation might be promising. The atypical dipeptide GAL-201 is orally bioavailable and interferes as a modulator of Aβ aggregation. It binds to aggregation-prone, misfolded Aβ monomers with high selectivity and affinity, thereby preventing the formation of toxic oligomers. Here, we demonstrate that the previously observed protective effect of GAL-201 on synaptic plasticity occurs irrespective of shortages and post-translational modifications (tested isoforms: Aβ_1–42_, Aβ(p3-42), Aβ_1–40_ and 3NTyr(10)-Aβ). Interestingly, the neuroprotective activity of a single dose of GAL-201 was still present after one week and correlated with a prevention of Aβ-induced spine loss. Furthermore, we could observe beneficial effects on spine morphology as well as the significantly reduced activation of proinflammatory microglia and astrocytes in the presence of an Aβ_1–42_-derived toxicity. In line with these in vitro data, GAL-201 additionally improved hippocampus-dependent spatial learning in the “tgArcSwe” AD mouse model after a single subcutaneous administration. By this means, we observed changes in the deposition pattern: through the clustering of misfolded monomers as off-pathway non-toxic Aβ agglomerates, toxic oligomers are removed. Our results are in line with previously collected preclinical data and warrant the initiation of Investigational New Drug (IND)-enabling studies for GAL-201. By demonstrating the highly efficient detoxification of β-sheet monomers, leading to the neutralization of Aβ oligomer toxicity, GAL-201 represents a promising drug candidate against Aβ-derived pathophysiology present in AD.

## 1. Introduction

The global incidence of Alzheimer’s Disease (AD) is rapidly increasing, highlighting the urgent need for better causal therapies. In addition to the predominant pathologies involving Amyloid-β (Aβ) [1] and tau-protein [2,3], numerous alternative pathomechanisms have been proposed. But as Aβ pathology was first documented more than a century ago [4], many therapeutic approaches focus on this particular mechanism. For a long time, only symptomatic treatment was available, but with the approval of the antibody Lecanemab [5], Aβ oligomers and protofibrils came into focus as FDA-approved drug targets. Soluble Aβ oligomers are considered the primary toxic species in AD [6,7,8], and their presence is correlated with cognitive decline [9,10]. This explains why previous antibodies targeting only insoluble forms of Aβ (e.g., Gantenerumab) did not show a clinical benefit. Furthermore, it is theorized that disassociation from plaques or fibrils even increases the number of toxic oligomers [11]. An exception is the recently approved antibody Donanemab. It specifically targets Aβ(p3-42) in plaques [12] that acts as a seed for further aggregation [13]. On the contrary, the inhibition of Aβ formation through β- and γ-secretase inhibitors was not efficient in clinical trials and, moreover, was associated with severe side effects. The reason for this might be that Aβ monomers in physiological, picomolar concentrations are necessary for modulating synaptic plasticity [14]. These harmless forms have a random coil structure, whereas a conformational change into a β-sheet structure promotes aggregation and therefore leads to toxic levels of aggregated Aβ [15,16].

GAL-201 is the pharmacokinetically advanced follow-up compound of GAL-101 (originally MRZ-99030). GAL-101 has been intensively characterized in previous work: it is a small peptide, targeting aggregation-prone, misfolded (β-sheet structure) Aβ monomers with high selectivity and affinity. It induces a conformational change which allows the misfolded Aβ monomers to aggregate into non-toxic, amorphous clusters [17], thereby producing detoxification [18]. Both molecules promote a detoxification process following a self-replicating mechanism, previously described for GAL-101 by Rammes and Parsons [18] and Russ [19] as well as for GAL-201 by Russ [20]. It is theorized that once the compound binds to the misfolded Aβ monomer, a conformational change is induced, which is then transferred to other misfolded peptides through a “prion-like seeding mechanism” [18,20]. In this process, the GAL-201-modulated monomer interacts with another misfolded monomer, causing it to adopt the same altered form. These two monomers can then associate with a third, and the cycle continues. Over time, this leads to the accumulation of Aβ monomers with a new, non-β-sheet structure [17,18,20]. This mode of action could be therapeutically promising, as it not only counters the disease process but does so by repurposing the mechanism that drives pathology. Aβ itself behaves like a prion, as is clearly presented by Ugalde [21]. Misfolded proteins in this context act as a template for further misfolding, meaning that each misfolded monomer promotes the same abnormal structure in others, leading to the formation of toxic oligomers [21]. In the presence of GAL-201, however, this prion-like replicating process results in structurally altered monomers that do not contribute to these harmful aggregates [17,18,20]. Our hypothesis is strongly supported by LTP (long-term potentiation) experiments with a serial dilution of GAL-201. In the experiments, the concentration of GAL-201 was far below any pharmacological activity per se, but the solution was still able to prevent and even reverse the synaptotoxic effects on CA1-LTP (long-term potentiation in *cornu ammonis* 1) induced by Aβ_1–42_, thereby strongly supporting the idea of a self-replicating mechanism where only initial pharmacological active concentrations are necessary to induce the self-replicating effect [20]. Through the binding of the misfolded Aβ monomers, the formation of various toxic Aβ aggregates such as dimers, trimers and oligomers is prevented, making GAL-201 highly efficient. Additionally, due to its high selectivity and affinity for the misfolded Aβ monomers, harmless, normally folded Aβ monomers, crucial for promoting LTP [14], are not targeted.

Previously, we have shown that GAL-201 triggers a self-propagating, self-detoxifying conformation of Aβ_1–42_ when a serial dilution was applied immediately after preparation [20]. In the present study, we intended to elucidate a possible time course of this protective effect. Therefore, serial dilution was applied with different time delays after preparation. Due to their abundance in AD-affected brains and their cellular toxicity, we investigated the beneficial prion-like effect against the synaptotoxicity of the isoforms Aβ_1–42_, Aβ(p3-42), Aβ_1–40_ and 3NTyr(10)-Aβ by performing in vitro CA1-LTP and analyzing spine dynamics, as well as by measuring various markers of glial neuroinflammation. As Aβ_1–40_ is, at approximately 90%, the most abundant isoform in the brain (vs. 5–10% Aβ_1–42_ and other subtypes) [22] and as Aβ_1–42_ is more aggregation-prone and most prominent in senile plaques [23], we decided to test both subtypes. In addition, as not only increased C-terminal length but also post-translational modifications increase toxicity, we had interest in testing these as well. Aβ(p3-42) is, among the N-terminal-truncated peptides, the most present form in senile plaques (>50%) [24] and works as a promotor for further aggregation, which applies to 3NTyr(10)-Aβ as well. This subtype is also primarily found in the plaque cores and accelerates aggregation [13,25]. As Aβ(p3-42) became an FDA-approved target through the approval of Donanemab, we decided to investigate these subtypes as well. Moreover, we tested cognitive performance in an AD mouse model after an acute treatment with GAL-201 and visualized plaque formation afterward.

## 2. Results

### 2.1. Electrophysiology in Hippocampal Brain Slices

**Figure 1:**
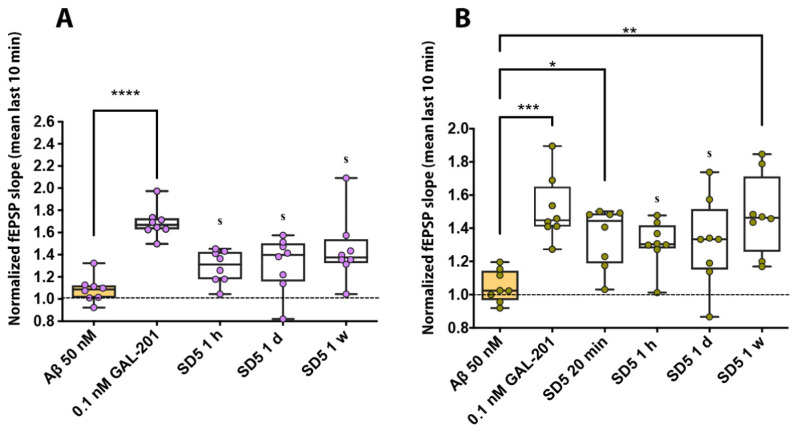

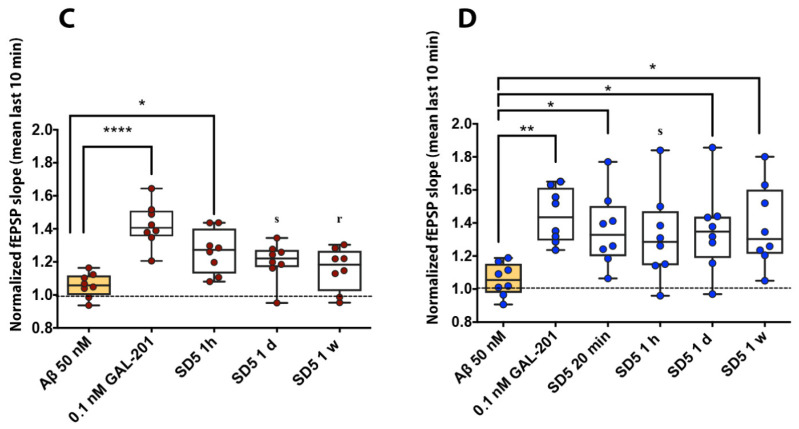
GAL-201 prevented detrimental effects of different Aβ subspecies on CA1-LTP—even after serial dilution. CA1-LTP was blocked when hippocampal brain slices were incubated with 50 nM of either (**A**) Aβ_1–42_, (**B**) Aβ_1–40_, (**C**) Aβ(p3-42) or (**D**) 3NTyr(10)-Aβ. When a serial dilution of GAL-201 was simultaneously applied, this effect was prevented. LTP remained inducible—even one week after the preparation of the solution [statistics—Dunn’s multiple comparisons: * *p* < 0.05, ** *p* < 0.01, *** *p* < 0.001 and **** *p* < 0.0001; effect size (calculated as AUC-ROC): s = significant effect (AUC < 0.3 or >0.7 plus AUCCi excluding 0.5) and r = relevant effect (AUC < 0.3 or >0.7)].

At a concentration of 50 nM, Aβ_1–42_, Aβ_1–40_, Aβ(p3-42) and 3NTyr(10)-Aβ significantly blocked CA1-LTP. Compared to the control with 0.1 nM GAL-201, LTP was significantly decreased. Recently, we demonstrated that in the presence of serially diluted GAL-201 (SD5), the synaptotoxic effects of Aβ_1–42_ and Aβ(p3-42) on LTP were prevented. LTP was still inducible, which was substantiated by the significant difference between the freshly prepared GAL-201/Aβ mixture (incubation time: 20 min) and 50 nM Aβ alone [20]. In the present study, we could show these beneficial effects when using Aβ_1–40_ and 3NTyr(10)-Aβ. Most interestingly, the beneficial effect on LTP was still observable one week after the preparation of the serial dilution. Serial dilution with each examined Aβ subtype differed significantly from 50 nM Aβ with any time delay when looking at LTP inducibility (Figure 1, Table 1).

### 2.2. Analysis of Spine Dynamics

#### 2.2.1. Spine Density

**Figure 2:**
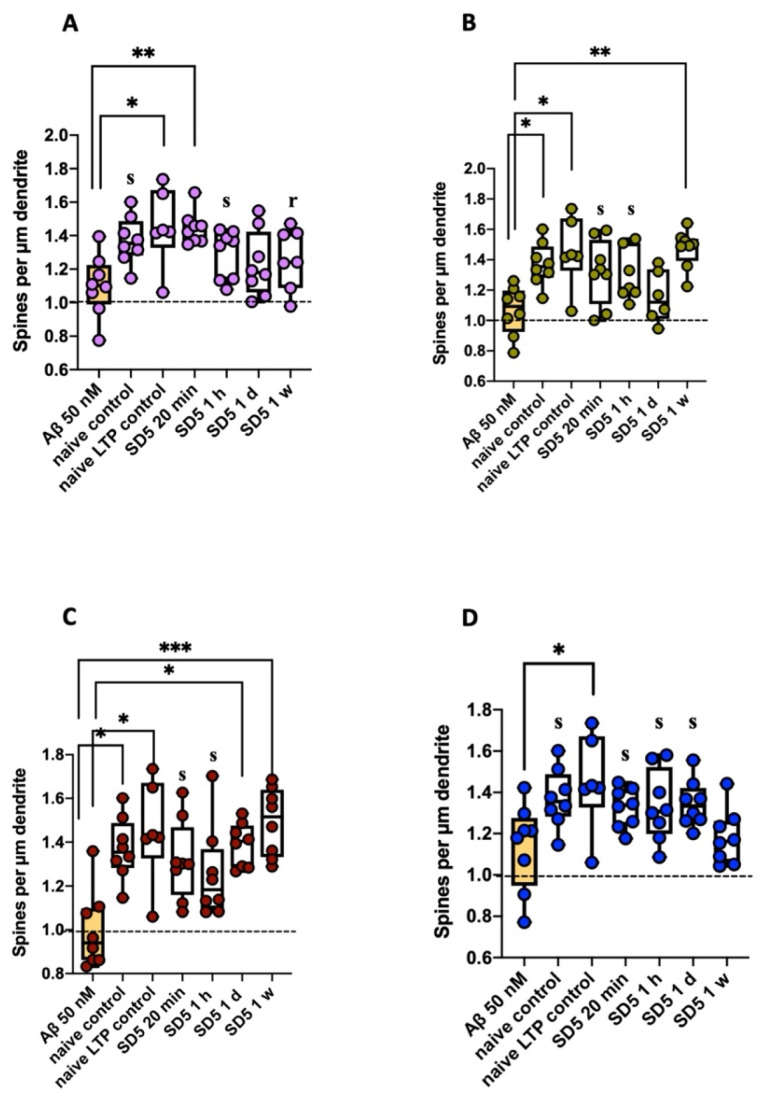
The beneficial effects of GAL-201 on LTP inducibility directly correlated with the prevention of Aβ-induced spine loss. Hippocampal brain slices which could not produce LTP because of (**A**) Aβ_1–42_-, (**B**) Aβ_1–40_-, (**C**) Aβ(p3-42)- and (**D**) 3NTyr(10)-Aβ-induced toxicity also showed a significant decrease in spines on their dendrites. Spine density was significantly reduced when compared to naive and naive LTP controls. However, if serial dilution of GAL-201 was simultaneously applied, Aβ-induced spine loss was prevented. This was shown for nearly every time delay with each Aβ subtype [only exceptions: GAL-201/Aβ1–42SD5 (1 d), GAL-201/Aβ1–40SD5 (1 d) and GAL-201/3NTyr(10)-AβSD5 (1 w)]. As the same tissue which was used in the LTP experiments earlier was used here, the results directly correlate with each other [statistics—Dunn’s multiple comparisons: * *p* < 0.05, ** *p* < 0.01 and *** *p* < 0.001; effect size (calculated as AUC-ROC): s = significant effect (AUC < 0.3 or >0.7 plus AUCCi excluding 0.5) and r = relevant effect (AUC < 0.3 or >0.7)].

**Figure 3:**
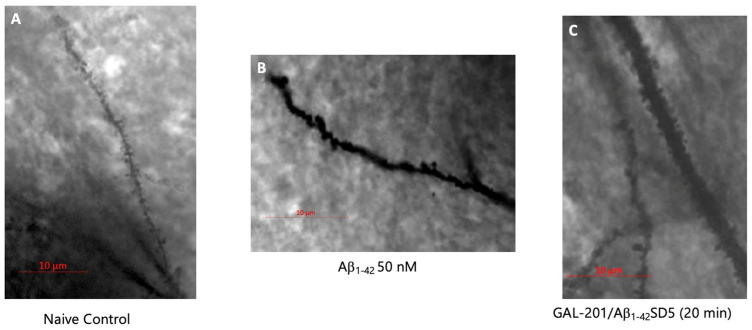
Aβ_1–42_-induced spine loss was visually prevented by GAL-201. Images of dendrites (**A**) in the naive control, (**B**) after incubation with 50 nM Aβ_1–42_ and (**C**) after incubation with GAL-201/Aβ1–42SD5 (20 min), taken with a 63× oil immersion lens. After incubation with 50 nM Aβ_1–42_, spine density was visually reduced—this was not the case when GAL-201 was simultaneously applied. Here, spine density was comparable to naive control conditions.

Hippocampal brain slices which could not produce LTP because of Aβ-induced toxicity also formed significantly fewer spines on their dendrites. Here, spine density was significantly reduced when compared to the naive and naive LTP controls (slice which experienced one high-frequency stimulation). After incubation with GAL-201, this was not observed. Here, significantly more spines were formed compared to the incubation with 50 nM Aβ. This was shown for nearly every time delay with each Aβ subtype. The only exceptions were GAL-201/Aβ1–42SD5 (1 d), GAL-201/Aβ1–40SD5 (1 d) and GAL-201/3NTyr(10)-AβSD5 (1 w). Here, no relevant difference compared to 50 nM Aβ was observed (Figure 2 and Figure 3, Table 2).

#### 2.2.2. Spine Morphology (According to Risher [26])

**Figure 4:**
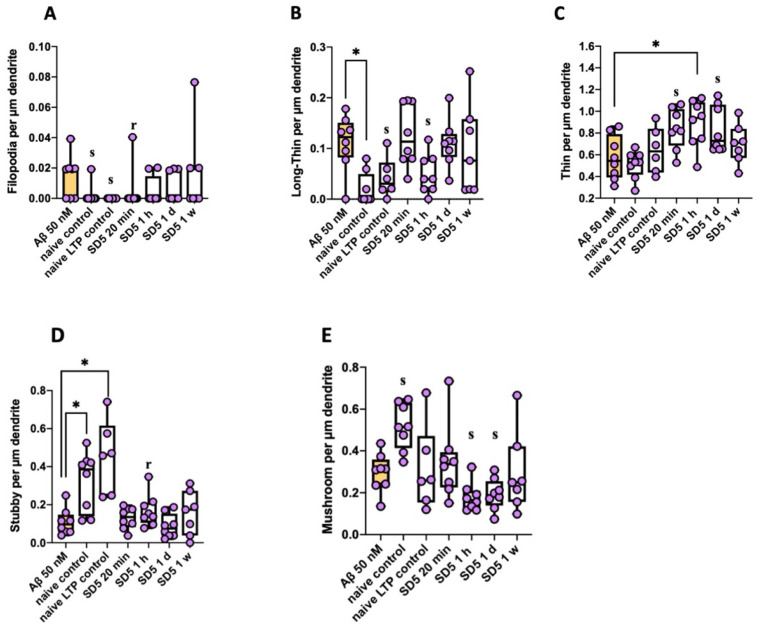
Spine morphology was altered by pharmacological treatment as well (example shown for Aβ_1–42_). (**A**) Filopodia spines, (**B**) long–thin spines, (**C**) thin spines, (**D**) stubby spines and (**E**) mushroom spines. In both controls, significantly fewer long spine types like filopodia or long–thin spines occurred compared to 50 nM Aβ_1–42_. Instead, stubby spines were significantly increased. In addition, the naive control displayed significantly more mushroom spines. These observations differed from an incubation with serially diluted GAL-201. Only slices of the one-hour time delay incubation displayed relevantly more stubby spines and significantly fewer long–thin spines, while after incubation with the 20 min dilution, significantly fewer filopodia spines occurred. However, after incubation with the 20 min dilution and one-hour and one-day time delay, significantly more thin spines were observed compared to 50 nM Aβ_1–42_ [statistics—Dunn’s multiple comparisons: * *p* < 0.05; effect size (calculated as AUC-ROC): s = significant effect (AUC < 0.3 or >0.7 plus AUCCi excluding 0.5) and r = relevant effect (AUC < 0.3 or >0.7)].

Spines were further characterized according to their morphology using the classification presented in Figure 1, by Risher [26]. Changes in spine morphology were observed, and examples are shown here for Aβ_1–42_. It was remarkable that after incubation with 50 nM Aβ_1–42_, significantly more longer spine types like filopodia or long–thin spines (immature, long spines required for new connections [27,28]) were present compared to control conditions. On the contrary, under control conditions, significantly more stubby spines (newly formed, short spines [29]) were formed. In addition, the naive control displayed significantly more mushroom spines (mature, broad spines with a high density of glutamate receptors [30]). This differed from incubation with serial dilution. Only after incubation with the one-hour time delay serial dilution was a relevant surplus of stubby spines present. Also, the density of filopodia and long–thin spines did not really differ from 50 nM Aβ_1–42._ Only after incubation with the fresh dilution were fewer filopodia formed, and after incubation with a one-hour time delay, fewer long–thin spines were formed. However, after incubation with fresh dilution (20 min) and a one-hour and one-day time delay, a significant surplus of thin spines (medium-length spines with high flexibility [30]) in comparison to 50 nM Aβ_1–42_ was observed (Figure 4, Table 3).

### 2.3. Activation of Microglia and Astrocytes

**Figure 5:**
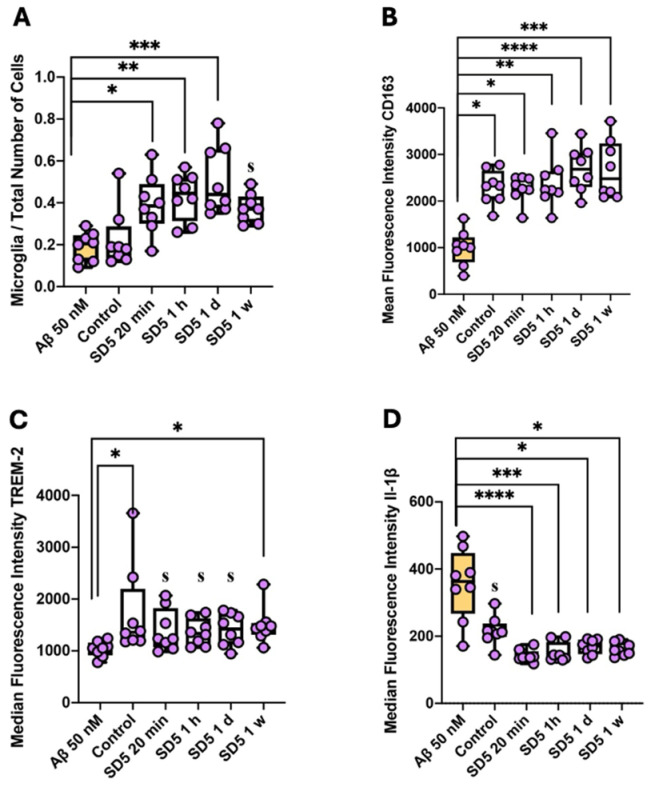
GAL-201 prevented Aβ_1–42_-induced microglia activation. (**A**) Microglia/total number of cells, (**B**) median fluorescence intensity of CD163, (**C**) median fluorescence intensity of TREM-2 and (**D**) median fluorescence intensity of IL-1β. When compared to 50 nM Aβ_1–42_, the relative microglia count was significantly higher after incubation with GAL-201. In addition, the expression of CD163 and TREM-2 was significantly increased, whereas the expression of IL-1β was significantly decreased, when compared to 50 nM Aβ_1–42_. Similar expression levels were observed under control conditions [statistics—Dunn’s multiple comparisons: * *p* < 0.05, ** *p* < 0.01, *** *p* < 0.001 and **** *p* < 0.0001; effect size (calculated as AUC-ROC): s = significant effect (AUC < 0.3 or >0.7 plus AUCCi excluding 0.5)].

Following the administration of Aβ_1–42_, we documented a notable increase in microglia within the experimental sample (digested hippocampal slice). Specifically, there was a doubling of the relative microglial density (percentage of the total cell count) observed after incubation with the 20 min post-preparation dilution series, with these elevated levels persisting even with a one-week time delay (Figure 5A). This finding indicates a rapid and sustained microglial response to GAL-201/Aβ1–42SD5 exposure. Despite this general increase in microglial numbers, the expression profiles of specific microglial markers showed differential responses after incubation with dilution series. Notably, the levels of CD163 (Cluster of Differentiation 163), a receptor involved in anti-inflammatory responses within the brain, remained unchanged when compared to control samples, whereas samples incubated with Aβ_1–42_ only showed significantly decreased levels of CD163 (Figure 5B). Similarly, the expression of triggering receptor expressed on myeloid cells 2 (TREM-2), which plays a key role in microglial phagocytic activity, was also unaltered compared to the control and decreased after Aβ_1–42_ exposure only (Figure 5C). In contrast with the increased levels of CD163 and TREM-2 expression, we observed a significant immunosuppressive effect on microglia, as evidenced by the significantly lower production of interleukin-1 beta (IL-1β) in the control samples as well as in the samples incubated with GAL-201 (Figure 5D). IL-1β is a pro-inflammatory cytokine predominantly produced by activated microglia and is crucial for initiating and propagating inflammatory responses in the central nervous system. So, compared to Aβ exposure only, IL-1β expression levels were significantly decreased when simultaneously incubated with GAL-201, suggesting a shift toward an immunosuppressed state of microglia, despite an overall increase in their numbers. This could indicate a compensatory, potentially protective microglia response in the presence of GAL-201 aimed at mitigating excessive inflammation following Aβ_1–42_ (Figure 5, Table 4).

**Figure 6:**
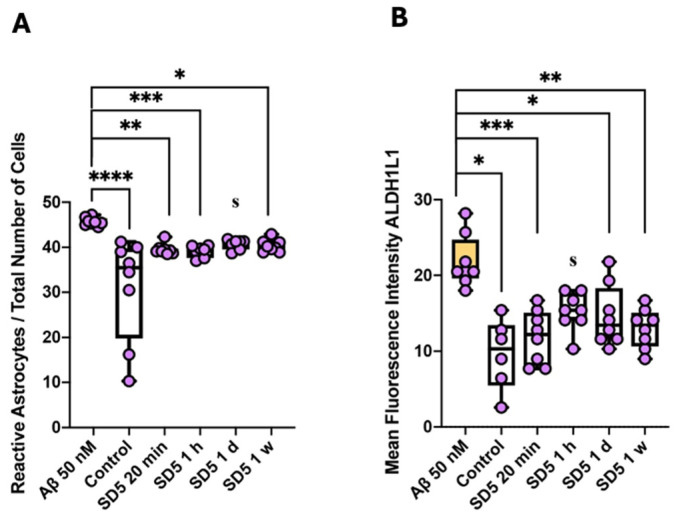
GAL-201 also prevented Aβ_1–42_-induced astrocyte activation. (**A**) Reactive astrocytes/total number of cells and (**B**) median fluorescence intensity of ALDH1L1. Compared to control conditions or incubation with GAL-201, the relative count of reactive astrocytes was significantly increased after incubation with 50 nM Aβ_1–42_. In addition, reactive astrocytes expressed significantly lower levels of ALDH1L1 under control conditions and after incubation with GAL-201 than after incubation with 50 nM Aβ_1–42_ [statistics: Dunn’s multiple comparisons: * *p* < 0.05, ** *p* < 0.01, *** *p* < 0.001 and **** *p* < 0.0001; effect size (calculated as AUC-ROC): s = significant effect (AUC < 0.3 or >0.7 plus AUCCi excluding 0.5)].

The relative count of reactive astrocytes (GFAP-positive cells = glial fibrillary acidic protein) was significantly increased compared to control conditions after incubation with 50 nM Aβ_1–42_, indicating Aβ_1–42_-induced astrocyte activation. On the contrary, similar percentages to control conditions were observed after incubation with GAL-201, suggesting a protective effect of GAL-201 against Aβ_1–42_-induced astrocyte activation. In addition, the ALDH1L1 (aldehyde dehydrogenase 1 family member L1) expression level of reactive astrocytes significantly increased after incubation with 50 nM Aβ_1–42_, whereas significantly less ALDH1L1 was expressed under control conditions as well as after incubation with GAL-201, supporting the observed effect on astrocyte activation (Figure 6, Table 4).

### 2.4. Cognitive Performance Was Improved in tgArcSwe Mice After Acute Treatment with GAL-201

**Figure 7:**
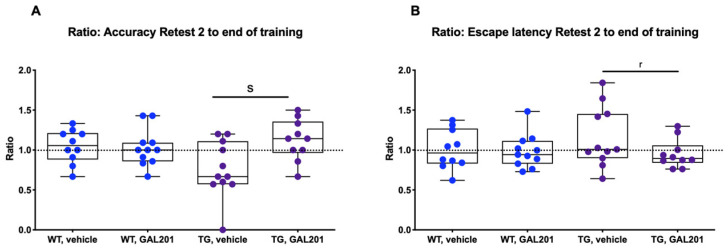
Beneficial effects of GAL-201 on hippocampus-dependent spatial learning shown by WCM behavioral tests. (**A**) Accuracy and (**B**) escape latency: Each treatment group was compared to its corresponding vehicle group. There was no multiple comparisons test between WT and TG. The TG treatment group demonstrated a significantly higher accuracy in retest 2 than the vehicle-treated control. In addition, the TG treatment group showed a relevantly improved escape latency in retest 2 when compared to the vehicle group [statistics—effect size (calculated as AUC-ROC): s = significant effect (AUC < 0.3 or >0.7 plus AUCCi excluding 0.5) and r = relevant effect (AUC < 0.3 or >0.7)].

To demonstrate the impact of GAL-201 on cognitive function in an AD mouse model, we tested cognitive performance in the WCM (Water Cross Maze). Here, the TG (transgenic) vehicle group performed worse than the WT (wildtype) vehicle group in terms of both escape latency (AUC = 0.62 [0.35; 0.86]) and accuracy (AUC = 0.24 [0.06; 0.47]). Each treatment group was compared to the corresponding vehicle group. There was no difference in accuracy between the TG treatment group and the vehicle group on training day 5 (AUC = 0.51 [0.28; 0.75]). Nevertheless, at retest 1, a relevant difference in accuracy between the TG treatment and vehicle group compared to their respective accuracy at the end of the training phase was observed, as evidenced by the effect size (AUC = 0.69 [0.44; 0.92]). This difference became significant in retest 2 (AUC = 0.8 [0.59; 0.96]). In addition, retest 2 showed an almost significantly faster escape latency for TG mice with GAL-201 treatment (AUC = 0.3 [0.08; 0.55]). At retest 2, the WT groups showed a relevant difference neither in accuracy (AUC = 0.45 [0.19; 0.69]) nor in escape latency (AUC = 0.48 [0.22; 0.74]) (Figure 7).

### 2.5. Changes in the Occurrence of Plaques in tgArcSwe Mice After Acute Treatment with GAL-201

**Figure 8:**
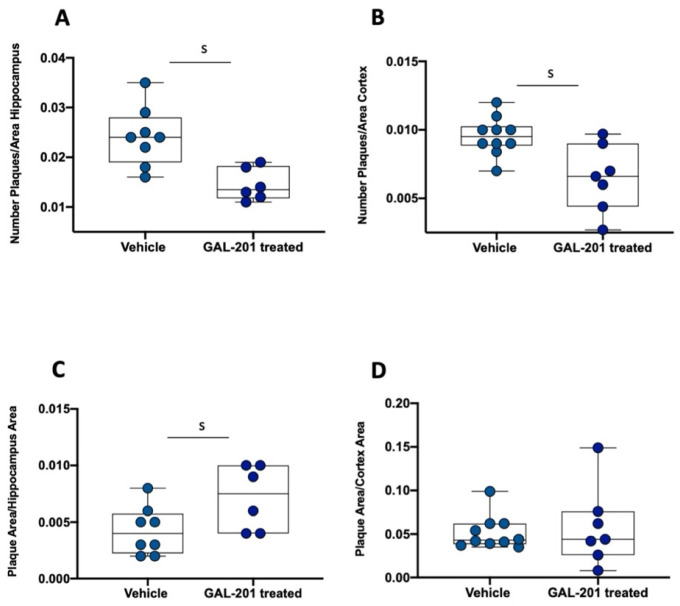
GAL-201 reduced the number of plaques while increasing the plaque area. (**A**) Number of plaques/area of hippocampus and (**B**) number of plaques/area of cortex: following a single administration of GAL-201, the number of plaques relative to the total hippocampal/cortical area significantly decreased. (**C**) Plaque area/hippocampus area and (**D**) plaque area/cortex area: conversely, the plaque area relative to the hippocampal area significantly increased. No significant difference was observed when the plaque area was correlated with the total cortical area [statistics—effect size (calculated as AUC-ROC): s = significant effect (AUC < 0.3 or >0.7 plus AUCCi excluding 0.5).

Following acute treatment with GAL-201 in tgArcSwe mice, the number of Aβ plaques in the mouse brain relative to the total hippocampal/cortical area significantly decreased (AUC = 0.93 [0.75; 1]/AUC = 0.87 [0.66; 1]) (Figure 8A,B). On the contrary, the plaque area relative to the hippocampal area significantly increased (AUC = 0.20 [0; 0.48]). No significant difference was observed when the plaque area was correlated with the total cortical area (AUC = 0.47 [0.17; 0.78]) (Figure 8C,D).

## 3. Discussion

### 3.1. Consistency of the Results

Through the use of an LTP assay, GAL-201 has previously demonstrated efficacy in reducing the synaptotoxic effects induced by Aβ_1–42_ oligomers, both in vitro and in vivo. Notably, it also exhibited the ability to reverse an ongoing toxic effect, positioning it as a promising drug candidate against AD [20]. Interestingly, the protection of the induction of LTP in the presence of different Aβ isoforms persisted even after the serial dilution of GAL-201 [20], and our findings indicate that this effect remains with time delays up to one week after preparation. We observed this phenomenon regarding the synaptotoxicity of all isoforms tested, Aβ_1–42_, Aβ_1–40_, Aβ(p3-42) and 3NTyr(10)-Aβ, which is likely due to the binding motif located in the hydrophobic core of the Aβ peptide (amino acids 16–20), which is not affected by these modifications. Even though the intensity of the beneficial impact differed among the Aβ subtypes, a significant difference in effect size was observed for all isoforms and all time delays. Thereby, the most linear effects were demonstrated for Aβ_1–42_ and Aβ(p3-42), with most prominent effects after applying fresh serial dilution [20], likely due to their accelerated aggregation behavior [31,32,33,34]. In contrast, for Aβ_1–40_ and 3NTyr(10)-Aβ the observed effect was less linear with similar effects throughout all time delays (Figure 1). Furthermore, we found that the beneficial effect of GAL-201 is associated with the prevention of Aβ-induced spine loss (Figure 2 and Figure 3) and beneficial effects on spine morphology (Figure 4). Moreover, GAL-201 demonstrated protection against the Aβ_1–42_-induced activation of proinflammatory microglia (Figure 5) and astrocytes (Figure 6). These beneficial outcomes were further supported by an enhancement in cognitive performance observed in tgArcSwe mice following an acute treatment with GAL-201 (Figure 7), which also correlated with a decreased number of Aβ plaques relative to the total hippocampal/cortical area (Figure 8).

### 3.2. Interpretation of the Dilution Series

In GAL-201, the terminal carboxy group of its precursor GAL-101 is substituted with a terminal amide group. Through this alteration, the binding affinity to misfolded Aβ_1–42_ monomers is augmented [20], leading to the very efficient binding of these monomers. Previous experiments demonstrated that the highest efficacy in reducing the synaptotoxic effects on CA1-LTP induced by 50 nM Aβ_1–42_ was achieved following incubation with 10 nM GAL-201 [20]. In contrast, Aβ-aggregation inhibitors, e.g., ALZ-801, need much higher concentrations [35]. But the increased potency of GAL-201 can be attributed not only to an improved affinity and selectivity. We hypothesize that the prion-like seeding mechanism, first described for GAL-101 by Russ [19] and Rammes and Parsons [18], contributes to the high effectiveness of GAL-201, too. This mechanism is evidenced by experiments with a serial dilution of GAL-201. While keeping the Aβ concentration constant, GAL-201 was almost eliminated from the medium by stepwise dilution (final GAL-201 concentration was 0.1 nM). As this solution was still able to prevent Aβ_1–42_-induced neurotoxicity against CA1-LTP [20], this experiment strongly supports the prion-like seeding mechanism. Detoxification was likely initiated upon exposure to the peak concentration of GAL-201, applied in the first vial of the dilution series. By inducing a conformational change in misfolded Aβ monomers, GAL-201 may promote the formation of structurally altered monomers that act as templates for other misfolded species, propagating a non-amyloidogenic pathway. This process leads to the formation of non-toxic, amorphous clusters, as previously characterized by Parsons [17]. Importantly, this mechanism of action appears to be independent of sustained drug levels, which may explain why the serial dilution of GAL-201 remains effective in preventing the Aβ-induced impairment of CA1-LTP. Prior studies have confirmed that the observed effects on LTP are not attributable to the time-dependent degradation of Aβ toxicity, and that direct incubation with 0.1 nM GAL-201 alone does not elicit detoxification [20].

### 3.3. Influence on Spine Dynamics

The immediate effect of GAL-201 on LTP inducibility in the presence of Aβ directly correlated with the prevention of Aβ-induced spine loss. After incubation with 50 nM Aβ of any isoform tested, spine density significantly decreased compared to control conditions. However, incubation with GAL-201 prevented this effect (Figure 2). As the same slices subjected to LTP experiments were used for staining thereafter, the quantification of spines strongly correlates with the capability of LTP induction (see, e.g., Hayashi [36]). Through its mode of action, GAL-201 was able to prevent the Aβ-induced loss of spines and therefore maintained LTP inducibility. But not only spine density is altered by LTP induction; changes in spine morphology also occur within minutes and can be already observed 30 min after induction [37], which was confirmed in these results. Compared to control conditions, incubation with 50 nM Aβ_1–42_ led to the formation of significantly more longer spines like filopodia or long–thin spines. In turn, under control conditions, significantly more stubby spines were formed. Naive controls additionally showed a significant surplus of mushroom spines. Mushroom spines bearing enough space for the dynamic adaptation of AMPA receptors in case of LTP [38] are therefore described as “memory spines” [30]. Mushroom spines represent a mature spine type, which normally occur more often in later spine development [30]. This explains why they were most frequent under naive control conditions—no remodeling due to high-frequency stimulation or pharmacological treatment was induced here. On the contrary, filopodia, long–thin and stubby spines are early spine types [30], whereas filopodia and long–thin spines are used to find and initiate new connections between synapses [27,28]. Our findings indicate that Aβ_1–42_-induced spine loss was associated with a remodeling of the existing spines back to immature and longer spine types. With a few exceptions, incubation with serial dilution (all time delays) did not lead to this kind of remodeling. But as no decrease in spine density was observed here, other spine types must have been promoted. This was verified by evaluating thin spines. They can easily differentiate into other spine types and therefore are described as “learning spines” [30]. After incubation with GAL-201/Aβ1–42SD5 (20 min, 1 h, 1 d), these spines were significantly increased compared to 50 nM Aβ_1–42_, which led to the assumption that pharmacological treatment with GAL-201 might induce a learning process (Figure 4).

### 3.4. Impact on Behavior

Intriguingly, similar beneficial results were observed when TG mice, bearing an Aβ-derived pathophysiology, were subjected to an acute GAL-201 treatment before cognitive testing. TG mice that received a single subcutaneous injection of GAL-201 exhibited significantly improved spatial orientation in comparison to vehicle-treated animals 6 weeks after the injection. This implies that GAL-201-treated animals learned better than the vehicle-treated animals, which manifested in a significant difference 6 weeks later. Of course, this could be a temporary effect, and further experiments with longer time periods and chronic administration of GAL-201 are required to further investigate this effect. But, as according to pharmacokinetic data (elimination half-life of 2 h [20]) and after a single injection, it is unlikely that at the end of the training phase or at the final retest an active concentration of GAL-201 is still present in the brain, this first experiment strongly supports the self-replicating mechanism of GAL-201. The in vitro data clearly demonstrated a long-lasting, prion-like protective effect even one week after preparing the solution. Combining these results with those from cognitive testing, one may hypothesize that an acute dose of GAL-201 is able to initiate a detoxifying effect, which is effective over the whole period of the training phase. Since monomers and oligomers are in a dynamic equilibrium, the monomer-binding compound GAL-201 can effectively target even an ongoing pathology. As a result, TG animals were able to perform similarly to WT mice in retest 2, almost six weeks after treatment (Figure 7). This hypothesis is further supported by the occurrence of Aβ plaques in the brain of tgArcSwe mice. The number of plaques relative to both the hippocampal and cortical areas decreased, while the plaque area relative to the hippocampal area increased (the difference relative to the cortical area was not significant but showed a similar trend) (Figure 8). This clearly supports the idea of Aβ-aggregation modulation. Once GAL-201 binds to misfolded Aβ monomers, self-detoxification is induced by collecting additional misfolded monomers through a prion-like mechanism [19]. Consequently, amorphous, non-toxic, non-β-sheet clusters are formed [17]. So, instead of inhibiting Aβ aggregation, GAL-201 modulates this process by triggering a non-amyloidogenic detoxifying off-pathway, thereby reducing the amount of intermediate toxic soluble oligomeric Aβ species. This mechanism could explain the observed changes in plaque morphology after single-dose treatment with GAL-201 in an in vivo model.

### 3.5. Influence on Reactive Gliosis and Neuroinflammation

Reactive gliosis and neuroinflammation are hallmarks of AD and may actively contribute to disease progression and chronicity [39,40]. In the present study, we clearly demonstrated that, at least in the presence of Aβ_1–42_, GAL-201 exhibited anti-inflammatory effects. Compared to 50 nM Aβ_1–42_, the relative amount of microglia was significantly increased after incubation with GAL-201. This might be, at first glance, surprising, but the expression of CD163 and TREM-2 was also increased, while the expression of IL-1β was decreased, suggesting that the augmented count of microglia is due to an elevation in anti-inflammatory microglia (Figure 5). CD163 is a marker for anti-inflammatory microglia [41], and TREM-2 is a microglia receptor which is believed to initiate the differentiation of homeostatic microglia to disease-associated microglia (DAM) [42]. These DAM are initially anti-inflammatory (DAM-1), phagocyting Aβ and inhibiting the release of pro-inflammatory cytokines [43]. This explains why in an aged brain, TREM-2 expression is increased [41]. In contrast, in AD brains, TREM-2 was found to be less activated, which was related to a loss of protective effects [44]. Under these circumstances, Aβ, together with other endogenous ligands, can induce chronic overstimulation, which causes a transformation into pro-inflammatory DAM-2 [42]. On the contrary, IL-1β is a pro-inflammatory marker which is overexpressed in activated microglia (Figure 5) [39]. Regarding astrocyte activation, a similar result was observed. Compared to control conditions, the relative number of reactive astrocytes was significantly higher after incubation with 50 nM Aβ_1–42_. This was not observed after incubation with GAL-201. Here, the relative number of reactive astrocytes was similar to that in control conditions. These findings were further supported by the expression of ALDH1L1. ALDH1L1 was shown to be increased in AD-affected brains [45], which was confirmed in our results. Compared to control conditions, incubation with 50 nM Aβ_1–42_ led to a significant increase in ALDH1L1 levels. This was prevented when GAL-201 was simultaneously applied. ALDH1L1 levels here were similar to those in control conditions (Figure 6). These findings collectively highlight the complex dynamics of microglia and astrocyte activation and the differential regulation of inflammatory pathways following exposure to Aβ_1–42_. Further studies will be necessary to elucidate the mechanisms governing these changes and their implications for neuroinflammatory diseases, but these data clearly show that the detoxification process initiated with GAL-201 impacts neuroinflammation.

### 3.6. Summary

GAL-201 is under preclinical development as an orally available AD drug and features a promising mode of action against Aβ-derived pathologies including AD. The compound targets misfolded Aβ monomers with high selectivity and affinity, thereby preventing toxic aggregation at source while sparing normally folded monomers required for synaptic plasticity. Furthermore, it is assumed that GAL-201 seeds the self-replication of non-toxic Aβ aggregates, which might explain the long-lasting improved cognition in the AD mouse model after a single injection of GAL-201 6 weeks earlier. Additionally, we showed that GAL-201 targets multiple Aβ subtypes, which is of high importance due to their abundance and toxicity in the AD-affected brain. LTP was protected irrespective of the tested isoforms when a serial dilution of GAL-201 was applied, even one week after solution preparation. These findings correlated with the prevention of Aβ-induced spine loss and beneficial effects on spine morphology and strongly support the postulated self-replicating detoxification mechanism. Moreover, an influence on neuroinflammation was observed, which was consistently observed as an anti-inflammatory effect of GAL-201 in the presence of Aβ_1–42_. Due to its high affinity to misfolded Aβ, as well as the long-lasting biological effect, GAL-201 could become a potent oral drug, if human pharmacokinetics supports this as well. In addition, its long-lasting efficacy based on the prion-like phenomenon makes this drug beneficial particularly for the treatment of patients with AD, as detoxification is not dependent on constant drug levels and missed doses should not directly impact the treatment effect. These experiments represent a comprehensive extension of the data already published by Russ [20] and clearly warrant further development. The next step will be the initiation of IND-enabling studies with oral GAL-201.

## 4. Materials and Methods

### 4.1. Animals

For the in vitro experiments, 8- to 12-week-old male C57BL/6 mice (Charles River, Calco, Italy) (n = 8) were used. The mice were housed in climate-controlled cages at 23 ± 1 °C with a 12 h day/night rhythm and ad libitum access to water and food. For the behavioral experiment, we used mice from the tgArcSwe line carrying the Swedish mutation (K670N + M671L), which increases Aβ secretion [46], and the Arctic mutation (E693G), which increases Aβ protofibril formation [47,48]. Female wildtype (WT) mice were housed in groups of up to four, while transgenic (TG) mice were housed in groups of up to two. Male mice were housed individually. The mice were transferred to a separate room with a reversed day/night rhythm, identical to their previous living conditions, two weeks prior to the behavioral test. This enabled the experimenter to conduct the experiments during the day, but still within their active phase.

### 4.2. Brain Slice Preparation

For brain slice preparation, the mice were decapitated under isoflurane anesthesia and the brain was immediately transferred in an ice-cold Ringer solution (125 mM NaCl, 2.5 mM KCl, 25 mM D-glucose, 25 mM NaHCO_3_, h 1.25 mM NaH_2_PO_4_, 0.5 mM CaCl_2_, 6 mM MgCl_2_, pH 7.3—all Sigma-Aldrich, St. Louis, MO, USA) which was continuously carbogenated (95% O_2_/5% CO_2_). The brain was divided along the two hemispheres, which were then cut into 350 μm thick sagittal brain slices using a microtome (Microm HM 650V Thermo Fischer, Waltham, MA, USA). The hippocampus was isolated, and the slices were transferred into an aCSF-filled (artificial cerebrospinal fluid: 125 mM NaCl, 2.5 mM KCl, 25 mM D-glucose, 25 mM NaHCO_3_, 1.25 mM NaH_2_PO_4_, 2 mM CaCl_2_, 1 mM MgCl_2_, pH 7.3—all Sigma-Aldrich), carbogenated chamber where they recovered for 30 min at 37 °C and at least 60 min at room temperature (22–24 °C).

### 4.3. Electrophysiological Recording (Long-Term Potentiation)

After 90 min recovery time, one slice was chosen and transferred into the recording chamber which was continuously perfused with carbogenated aCSF (4 mL/min). It was fixed with two nylon filaments framed by a platinum wire. For measuring the field excitatory postsynaptic potentials (fEPSPs) of two non-overlapping populations of the Schaffer collaterals, two twisted, tungsten, bipolar electrodes (Hugo Sachs Elektronik Harvard Apparatus GmbH (March, Germany), tip diameter: 50 μm, insulated) were placed in the CA1 *stratum radiatum* as stimulating electrodes. Between them, the recording electrode was placed. For this, an aCSF-filled borosilicate glass micropipette (Hugo Sachs Elektronik Harvard Apparatus GmbH) with an open tip resistance of 1–2 MΩ was used. Stimulus intensity was set to a strength at which potentials with 25 to 30% of the maximum response were evoked, and a stimulus was delivered in an alternating rhythm every 15 s from either electrode. Two recordings were averaged so that there was only one data point for each electrode every minute. After measuring a stable baseline for at least 20 min, the first high-frequency stimulation (100 Hz) was delivered from one randomly chosen electrode and the response was recorded for 60 min. This served as an internal control for a healthy slice. In case of a later incubation with serial dilution, the slice was preincubated with 0.1 nM GAL-201 for 60 min. So, here, the first tetanic stimulation was not only a control for the healthiness of the slice, but it also additionally served as proof that 0.1 nM GAL-201 itself does not limit LTP. The slice was then incubated either with serial dilution or with 50 nM Aβ for 90 min. After this, the second high-frequency stimulation was delivered from the other electrode and the response was recorded for 60 min again. We measured the fEPSP slope between 20 and 80% of the peak response and normalized it according to the baseline afterward. Long-term potentiation (LTP) was defined as a slope that was equal to or more than 120% of baseline. The fEPSPs were amplified (BA-2S, npi electronic, Tamm, Germany), filtered (3 kHz) and digitalized (9 kHz) via a laboratory interface (ITC-16, Instrutech Corp., St Longmont, CO, USA) and the program WinLTP (version 2.4). For normalization, Excel was used, and for graphical analysis, GraphPad Prism (version 8, Dotmatics, Boston, MA, USA) was used. All experiments were conducted at room temperature, and previous experiments ensured that the extent of LTP is not dependent on time (up to five hours) [18].

### 4.4. Preparation of Amyloid-β, GAL-201 and Serial Dilution

#### 4.4.1. Amyloid-β

A total of 1 mg Aβ_1–42_ (HCl salt from BACHEM, CAS: 107761–42-2, batch: 1000039223) was dissolved in Hexafluoroisopropanol 100% (HFIP, Sigma-Aldrich) and incubated for 90 min at room temperature. Afterward, aliquots of 50 μg were prepared and HFIP was removed in a drying cabinet at 37 °C. The aliquots were then stored at −80 °C until use. For use, they were dissolved in dimethyl sulfoxide (DMSO) 99.9% (Sigma-Aldrich) to a concentration of 100 μM and then further diluted with aCSF to the desired concentration. The preparation of Aβ_1–40_ (salt: Hydrochloride, Bachem, Bubendorf, Switzerland, batch: 1066313) and Aβ(p3-42) (salt: Ammonium, Bachem, batch: 1061309) was conducted analogously to this process. For the preparation of 3NTyr(10)-Aβ, Aβ_1–42_ (salt: Hydrochloride, Bachem, batch: 1000039223) was dissolved in NaOH (Sigma-Aldrich), diluted with pi- buffer (according to Sörensen) and incubated with Peroxynitrate (Cayman, Ann Arbor, MI, USA) for one minute. This solution was also stored at −80 °C until use, but it already had a concentration of 100 μM and could directly be further diluted with aCSF.

#### 4.4.2. GAL-201

GAL-201 (C_15_H_20_N_4_O_2_, MW 288.34 g/Mol) was provided by Galimedix Therapeutics Inc. (Kensington, MD, USA) as a fumarate salt. GAL-201 was dissolved in MilliQ-H_2_O, and a 10 mM stock solution was prepared. From this, 50 μL aliquots of a 1 mM working solution were prepared via dilution with aCSF. For these experiments, the batch # QAF-10175 v1 of 3 December 2018 with 99.8% purity was used.

#### 4.4.3. Serial Dilution (GAL-201/AβSD5)

**Figure 9:**
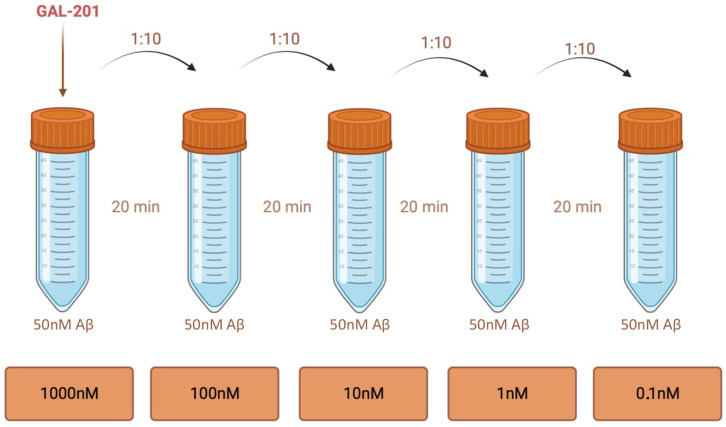
Serial dilution of GAL-201. While maintaining a constant concentration of 50 nM Aβ, GAL-201 was gradually diluted, starting from a concentration of 1000 nM in the first vial. After 20 min, GAL-201 was diluted 1:10. This step was repeated three more times, resulting in a final concentration of 0.1 nM GAL-201 (500:1 Aβ over GAL-201), a concentration far below target affinity and therefore any pharmacological activity. But as solution 5 has been shown to be no longer toxic in LTP experiments, it seems like, in the beginning, high concentrations of GAL-201 are necessary to initiate the prion-like mechanism. The bound peptides then act as a seed for further non-β-sheet aggregation. To investigate the time course of this protective effect, serial dilution was not only applied directly after preparation—the same dilution was also applied with a time delay of one hour, one day and one week after solution preparation. Created with BioRender.com.

The preparation of the serial dilution was conducted analogously to the methods of Rammes and Parsons [18] and Russ [20]. While maintaining a constant concentration of 50 nM Aβ, GAL-201 was diluted in four steps, starting with a concentration of 1000 nM in the first vial. After 20 min, GAL-201 was diluted 1:10. This step was repeated three more times, resulting in a 500:1 stoichiometric excess of Aβ over GAL-201 and a final concentration of 0.1 nM GAL-201. Serial dilution was applied with a time delay of 20 min (fresh dilution), but also with time delays of one hour, one day and one week after preparation (Figure 9).

### 4.5. Analysis of Spine Dynamics (Golgi Staining)

After finishing the LTP experiments, the slices were fixed overnight and impregnated the next day [note: as high-frequency stimulation itself induces spine remodeling [49,50], there were two controls—one naive slice (naive control) and one slice which was untreated but stimulated with 100 Hz (naive LTP control)]. The impregnation took seven days, after which the slices were Golgi-stained. For fixation, impregnation and staining, the “sliceGolgi Kit” from Bioenno (Cat. no. 003760), including the protocol, was used. When the slides were dry, in each slice, five dendrites from the CA1 region were chosen and a z-stack was generated using a 63× oil immersion lens (Apotom Zeiss–Axiocam 503 Mono, Zeiss, Oberkochen, Germany). The dendrites were focused on with the use of Zen 3.0 (blue edition). Then, Fiji (ImageJ2, version 2.9.0) was used to restrict the image sequence to the actual needed sequence and to optimize contrast (https://imagej.net/software/fiji/downloads, accessed on 14 September 2022). For the analysis of spine density and spine morphology, the “rapid Golgi analysis method” described by Risher [26] and the program SynapseWeb RECONSTRUCT (32-bit) (version 1.1.0.0, 2007, UTexas, Austin, TX, USA) were used. Data were imported into Excel (version 2016, Microsoft, Redmond, WA, USA) and analyzed using the template given by the authors. For graphical analysis, GraphPad Prism (version 8, Dotmatics) was used.

### 4.6. Activation of Microglia and Astrocytes (Fluorescence-Activated Cell Sorting)

Slices were incubated in a separate incubation chamber for 90 min (with the exception of the control slice). After this, tissue was digested with 1 mL digestion solution (EDTA + L-Lysin + 1× PBS buffer from Sigma Aldrich, and 1×PBS from 10× Dulbecco’s PanReacAppliChem, Darmstadt, Germany) and 10 μL Papain (from Carica Papaya, Sigma-Aldrich) through a 15 min water bath incubation at 37 °C and mechanical separation. The cell suspension was filtrated (70 µm Avantar VWR^®^ Cell Strainer, Radnor, PA, USA, Cat. No.: 732-2758), washed with 1 mL 1×PBS buffer and transferred into a fresh vial. It was centrifuged (10 min, 4 °C, 600× *g*) and resuspended in 500 μL freezing medium (DMEM (1×) + GlutaMax™-I mit 10% DMSO, gibco and Sigma-Aldrich). Afterward, it was stored at −20 °C until use. The day before measurement, samples were slowly thawed. They were centrifuged (10 min, 4 °C, 600× *g*) and washed with 1× PBS buffer twice. Afterward, they were fixed for 30 min at 4 °C with 200 μL FixPerm (BD Cytofix/Cytoperm™, BD Biosciences, Franklin Lakes, NJ, USA). Samples were then washed with PermWash (BD Cytofix/Cytoperm™, BD Biosciences) twice (5 min, 4 °C, 600× *g*). In the subsequent step, 50 μL of blocking solution (49 µL PermWash + 1 µL TrueStain FcX™ (anti-mouse CD16/32, BioLegend, San Diego, CA, USA)) was added to the cell pellet, which was then incubated for five minutes at 4 °C in the dark. Meanwhile, antibody master mix (Table 5) was freshly prepared and added to each sample, which was then incubated overnight at 4 °C in the dark. The next day, samples were washed with PermWash again (5 min, 4 °C, 600× *g*) and resuspended with FACS fixation buffer (50% FACS buffer + 50% PFA 4%, Merck). Finally, samples were transferred into FACS vials, and 200 μL FACS buffer (EDTA, Sigma-Aldrich + sodium azide, Merck + BSA, Roth + HBSS, gibco) was added. For measurement, BD LSRFortessa™, Software BD FACSDiva™ (Version 6.1.3), was used.

After the cells of interest had been determined, microglia were primarily gated on CD11b^+^/CD45^low-int^ cells [51,52] and astrocytes on CD11b^−^/CD45^−^ cells [53]. CD11b^+^/CD45^low-int^ cells were further gated on F4/80^+^ cells, which were subsequently gated on P2RY12^+^ and TMEM119^+^ [51]. F4/80 is increased in hippocampal microglia [54] and in the brain, while TMEM119 is uniquely expressed by microglia [55,56]. P2RY12 is highly expressed by microglia as well, and it has been shown to colocalize with CD11b too [57]. Therefore, F4/80, P2RY12 and TMEM119 represent microglia-specific markers which were used to ensure that only microglia and no other monocytes were considered here. CD11b^−^/CD45^−^ cells were subsequently gated on GFAP^+^. GFAP is specific to astrocytes and increased in reactive ones [58]. As it does not cover all types of astrocytes, for differentiation, ALDH1L1 should be considered, too [59,60]. ALDH1L1 detects resting astrocytes as well, and gating on ALDH1L1^+^/GFAP^−^ can be used to identify them, whereas ALDH1L1^+^/GFAP^+^ cells represent reactive astrocytes [60]. As our samples had too low ALDH1L1 expression levels and mainly reactive astrocytes were of interest, samples were further gated only on GFAP^+^ cells. Thus, CD11b^+^/CD45^low-int^/F4/80^+^/P2RY12^+^/TMEM119^+^ cells represented microglia and CD11b^−^/CD45^−^/GFAP^+^ cells represented reactive astrocytes. Additionally, in both groups, the median fluorescence intensity of different activation markers was measured. The expression levels of CD163, TREM-2 and IL-1β were determined for microglia, whereas for reactive astrocytes, the expression level of ALDH1L1 was considered. This was because ALDH1L1 was found to be increased in AD, too [45]. All the antibodies that were employed are listed in Table 1. For gating, FlowJo (version 9.0, BD Biosciences) was used, and data were imported into Excel (version 2016, Microsoft) and processed graphically with GraphPad Prism (version 8, Dotmatics).

### 4.7. Behavioral Test

#### 4.7.1. Preparation and Administration of GAL-201

For the behavioral test, mice were assigned to four groups (wildtype/transgenic–treatment/vehicle) of 10 mice each (5 male, 5 female). GAL-201 was dissolved in MilliQ-H_2_O at a concentration of 8 mg/mL. The treatment group received a subcutaneous injection of GAL-201 at a dose of 80 mg/kg on the day before the training started, while the vehicle group received a subcutaneous injection of MilliQ-H_2_O at the same volume (0.01 mL/g).

#### 4.7.2. Water Cross Maze

**Figure 10:**
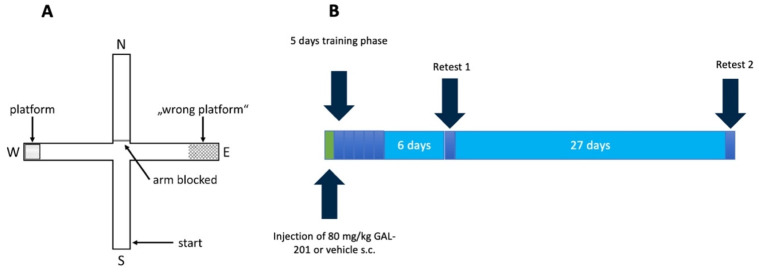
Test protocol for Water Cross Maze (WCM). (**A**) The WCM consisted of four Plexiglas arms, which formed a plus shape. It was surrounded by enough clues, and the initial position of the mouse was either in the northern or the southern arm—each opposite arm was blocked with a Plexiglas panel. The eastern arm contained a platform 1 cm below the water surface invisible to the mouse. (**B**) One day before the beginning of the training phase, the animals were injected s.c. with either GAL-201 or vehicle. On five consecutive days, the mouse underwent six WCM trials per day, alternating daily in an SNNSSN or NSSNNS starting pattern. Retest 1 was then conducted after one week—retest 2 was conducted four weeks after retest 1.

The Water Cross Maze (WCM) is a highly sensitive and well-established behavioral test for investigating hippocampus-dependent spatial learning [61]. It consists of four arms, each 50 cm long and 30 cm high, made of transparent Plexiglas, which form a plus shape and are named analogously to the four cardinal directions [61]. At the end of the western arm, a platform, 10 cm high and 8 × 8 cm in size, was placed [61]. The WCM was then filled with water to a height of 11 cm. The water was changed every day (water and room temperature: 22 °C). The experiment was conducted in a darkened room with a brightness of <15 lux [62]. A sufficient number of objects was placed around the WCM for orientation [61]. The experiment was conducted analogously to that of Kleinknecht [61]. The mouse started at either the northern or southern arm facing the experimenter. The opposite arm was blocked with a removable Plexiglas disk, forming a T-shape to facilitate the experiment and to reduce stress for the animal [61]. The experimenter stood approximately 20 cm behind the starting arm [61]. The experiment began when the mouse was placed in the water and ended when it reached the platform. A waiting period of 5 s was required before the mouse was retrieved by the examiner and returned to its cage. If the mouse left the platform before the 5 s were over, the timer was restarted. If the mouse did not find the platform, the experiment ended after a maximum of 60 s. After an experiment, the water was stirred with a plastic stick and the walls were cleaned from water droplets. Only water served as a negative reinforcement incentive. Therefore, the WCM is independent of other potentially unreliable rewards, such as food [61]. Six test runs were carried out per day, and the starting arms alternated in an “NSSNNS” or “SNNSSN” pattern. The mouse underwent a training phase of five consecutive days, followed by a one-week pause before the first retest. After an additional four weeks, the second retest was conducted (Figure 10).

The following variables were analyzed:

- Escape latency: time the mouse needed to reach the platform.
- Accuracy: percentage of trials per day in which the mouse found the platform directly without entering other arms.

An arm or platform was considered entered or reached when the mouse’s entire body, excluding its tail, was inside/on it [61]. The retest results were normalized with the mean value of the results from training days 4 and 5. Values above 1 indicated either a prolongation of escape latency or a higher accuracy.

### 4.8. Visualization of Plaque Formation (Methoxy-X04 Staining)

After retest 2 of the WCM, the animals were sacrificed on the same day by decapitation under deep isoflurane anesthesia. The brain was quickly dissected after decapitation, the cerebellum was discarded, and the hemispheres were separated. The hemispheres were then snap-frozen using liquid nitrogen or by placing them on a metal plate cooled with dry ice. The samples were stored at −80 °C until use. For the methoxy-X04 staining, one of the hemispheres was coronally cut into 70 µm thick slices using a cryotome (Thermo Scientific CryoStar NX70). The temperature of the blade was set to −18 °C and the chamber temperature to −14 °C. The slices were transferred onto glass slides and stored at −20 °C until staining. Before staining, the slides were washed with a 1:1 mixture of acetone and isopropanol (Sigma-Aldrich) for 20 min followed by a 1:1 mixture of 1× PBS (from 10× Dulbecco’s, PanReacAppliChem) and ethanol (96% Otto Fischar) for 10 min twice. The slides were then immersed in methoxy-X04 staining solution (TOCRIS) and incubated for 30 min in the dark on a 3D shaker. Afterward, they were washed thrice with 1× PBS/ethanol mixture (1:1) for 7.5 min and thrice with MilliQ-H_2_O for 10 min. The slides were then air-dried, covered with Fluorescence Mounting Medium (Dako, Santa Clara, CA, USA) and stored at 4 °C in the dark. For image processing, an ApoTom (Apotom Zeiss–Axiocam 503 Mono) with a 4′,6-diamidino-2-phenylindole filter and Zen 3.0 (blue edition) was used. After an overview image was taken at 2.5× magnification and the brain section was captured in its full extent, the magnification was switched to 10×, the focus was adjusted accordingly, and the section was then imaged in full size. The JPEG files were then transferred to ImageJ2, version 2.9.0) (https://imagej.net/software/fiji/downloads, accessed on 14 September 2022), where brightness and contrast were adjusted. Afterward, both the hippocampus and cortex were selected, the background outside of the selected area was removed, and the plaques were counted. Also, the plaque area, the surface area of the respective area, was measured to calculate the percentage of plaque coverage in relation to the total area.

### 4.9. Statistics

For statistical analysis, a non-parametric test (Kruskal–Wallis test) with a subsequent Dunn’s multiple comparisons test was performed, using GraphPad Prism (version 8, Dotmatics). In consideration of Rothman [63], there was no correction of multiple comparisons. Instead, the effect size as “area under the receiver operating characteristic” (AUC) with a 95% confidence interval (AUCCi) was calculated using the MES (=measures of effect size) MATLAB (Version: 9.6.0.1472908 (R2019a) Update 9) toolbox described by Hentschke and Stuttgen [64]. We utilized effect size measurements as they offer a more authentic depiction of the data, particularly when dealing with small biological sample sizes. They describe the difference between two groups. An AUC of 0.5 indicates no distinction between two groups, whereas an AUC < 0.3 and >0.7 suggests a relevant difference [65]. If the confidence interval excludes zero (AUC = 0.5), this is further defined as a significant difference [64]. Here, uncorrected *p*-values are marked with * for *p* < 0.05, ** for *p* < 0.01, *** for *p* < 0.001 and **** for *p* < 0.0001. Significant differences in effect size are marked as “s” and relevant differences in effect size as “r”. The AUC’s 95% confidence interval is presented in [ ].

## Figures and Tables

**Table 1:** Electrophysiological experiments.

	Control	SD5 (20 min)	SD5 (1 h)	SD5 (1 d)	SD5 (1 w)
Aβ_1–42_	*p* < 0.0001 with AUC = 0 [0; 0]	-	AUC = 0.13 [0; 0.33]	AUC = 0.16 [0; 0.42]	AUC = 0.09 [0; 0.28]
Aβ_1–40_	*p* < 0.001 with AUC = 0 [0; 0]	*p* < 0.05 with AUC = 0.06 [0; 0.2]	AUC = 0.08 [0; 0.28]	AUC = 0.1719 [0; 0.44]	*p* < 0.01 with AUC = 0.02 [0; 0.09]
AβpE3	*p* < 0.0001 with AUC = 0 [0; 0]	-	*p* < 0.05 with AUC = 0.08 [0; 0.25]	AUC = 0.13 [0; 0.38]	AUC = 0.23 [0; 0.53]
3NTyr(10)-Aβ	*p* < 0.01 with AUC = 0 [0; 0]	*p* < 0.05 with AUC = 0.08 [0; 0.25]	AUC = 0.17 [0; 0.43]	*p* < 0.05 with AUC = 0.13 [0; 0.34]	*p* < 0.05 with AUC = 0.06 [0; 0.23]

**Table 2:** Statistics for spine density.

	Control Naive	Control Naive LTP	SD5 (20 min)	SD5 (1 h)	SD5 (1 d)	SD5 (1 w)
Aβ_1–42_	AUC = 0.11 [0; 0.31]	*p* < 0.05 with AUC = 0.10 [0; 0.33]	*p* < 0.01 with AUC = 0.05 [0; 0.19]	AUC = 0.22 [0.03; 0.48]	AUC = 0.31 [0.08; 0.59]	AUC = 0.27 [0.04; 0.57]
Aβ_1–40_	*p* < 0.05 with AUC = 0.05 [0; 0.19]	AUC = 0.08 [0; 0.31]	AUC = 0.17 [0; 0.44]	AUC = 0.16 [0; 0.41]	AUC = 0.35 [0.08; 0.69]	*p* < 0.01 with AUC = 0.02 [0; 0.09]
AβpE3	*p* < 0.05 with AUC = 0.06 [0; 0.23]	AUC = 0.06 [0; 0.25]	AUC = 0.11 [0; 0.33]	AUC = 0.13 [0; 0.36]	*p* < 0.05 with AUC = 0.05 [0; 0.19]	*p* < 0.001 with AUC = 0.03 [0; 0.14]
3NTyr(10)-Aβ	AUC = 0.17 [0; 0.42]	*p* < 0.05 with AUC = 0.17 [0; 0.46]	AUC = 0.20 [0; 0.47]	AUC = 0.22 [0.03; 0.47]	AUC = 0.17 [0; 0.41]	AUC = 0.48 [0.19; 0.78]

**Table 3:** Statistics for spine morphology.

	Control Naive	Control Naive LTP	SD5 (20 min)	SD5 (1 h)	SD5 (1 d)	SD5 (1 w)
Filopodia	AUC = 0.77 [0.56; 0.94]	AUC = 0.81 [0.63; 0.94]	AUC = 0.71 [0.47; 0.94]	AUC = 0.82 [0.56; 1]	AUC = 0.68 [0.41; 0.91]	AUC = 0.5 [0.2; 0.8]
Long-Thin	AUC = 0.9 [0.7; 1]	AUC = 0.84 [0.58; 1]	AUC = 0.45 [0.17; 0.77]	AUC = 0.82 [0.56; 1]	AUC = 0.53 [0.23; 0.83]	AUC = 0.57 [0.25; 0.88]
Thin	AUC = 0.56 [0.25; 0.84]	AUC = 0.4 [0.13; 0.73]	AUC = 0.19 [0.02; 0.44]	*p* < 0.05 with AUC = 0.14 [0; 0.36]	AUC = 0.2 [0.02; 0.47]	AUC = 0.32 [0.07; 0.63]
Stubby	*p* < 0.05 with AUC = 0.08 [0; 0.23]	AUC = 0.04 [0; 0.17]	AUC = 0.41 [0.13; 0.7]	AUC = 0.27 [0.05; 0.55]	AUC = 0.61 [0.3; 0.89]	AUC = 0.39 [0.07; 0.73]
Mushroom	AUC = 0.05 [0; 0.17]	AUC = 0.54 [0.21; 0.88]	AUC = 0.41 [0.13; 0.7]	AUC = 0.84 [0.61; 1]	AUC = 0.84 [0.59; 1]	AUC = 0.59 [0.27; 0.89]

**Table 4:** Statistics for microglia and astrocyte activation.

	Control	SD5 (20 min)	SD5 (1 h)	SD5 (1 d)	SD5 (1 w)
Relative count microglia	AUC = 0.5 [0.2; 0.81]	*p* < 0.05 with AUC = 0.09 [0; 0.28]	*p* < 0.01 with AUC = 0.03 [0; 0.14]	*p* < 0.001 with AUC = 0 [0; 0]	AUC = 0.01 [0; 0.05]
CD163	*p* < 0.05 with AUC = 0 [0; 0]	*p* < 0.05 with AUC = 0 [0; 0]	*p* < 0.01 with AUC = 0 [0; 0]	*p* < 0.0001 with AUC = 0 [0; 0]	*p* < 0.001 with AUC = 0 [0; 0]
TREM-2	*p* < 0.05 with AUC = 0.06 [0; 0.22]	AUC = 0.22 [0.03; 0.48]	AUC = 0.11 [0; 0.31]	AUC = 0.16 [0; 0.39]	*p* < 0.05 with AUC = 0.05 [0; 0.19]
IL-1β	AUC = 0.86 [0.63; 1]	*p* < 0.0001 with AUC 0.98 [0.91; 1]	*p* < 0.001 with AUC = 0.97 [0.86; 1]	*p* < 0.05 with AUC = 0.94 [0.77; 1]	*p* < 0.05 with AUC = 0.95 [0.81; 1]
Relative count reactive astrocytes	*p* < 0.05 with AUC = 0.86 [0.64; 1]	*p* < 0.01 with AUC = 1 [1; 1]	*p* < 0.001 with AUC = 1 [1; 1]	AUC = 1 [1; 1]	*p* < 0.05 with AUC = 1 [1; 1]
ALDH1L1	*p* < 0.05 with AUC = 1 [1; 1]	*p* < 0.001 with AUC = 1 [1; 1]	AUC = 0.98 [0.93; 1]	*p* < 0.05 with AUC = 0.89 [0.67; 1]	*p* < 0.01 with AUC = 1 [1; 1]

**Table 5:** Antibodies used for fluorescence-activated cell sorting.

CD45	Brilliant Violet 510™ anti-mouse CD45, BioLegend (San Diego, CA, USA), CAT: 103137
CD11b	Brilliant Violet 785™ anti-mouse/human CD11b, BioLegend (San Diego, CA, USA), CAT: 101243
F4/80	PerCP/Cyanine5.5 anti-mouse F4/80, BioLegend (San Diego, CA, USA), CAT: 123127
TMEM119	Anti-Mo, TMEM119 PE_Cyanine7, Invitrogen (Waltham, MA, USA), CAT: 25-6119-80
P2RY12	APC anti-P2RY12, BioLegend (San Diego, CA, USA), CAT: 848005
CD163	Anti-Mo CD163 SuperBright™ 702, Invitrogen (Waltham, MA, USA), CAT: 67-1631-82
TREM-2	TREM-2 Monoclonal Antibody FITC, Invitrogen (Waltham, MA, USA), CAT: MA5-28223
IL-1β	IL-1Beta Rabbit anti-Mouse Polyclonal (aa118-269) (APC, Cy7) Antibody, LS Bio (Lynnwood, WA, USA), CAT: LS-C730762-100
GFAP	Mouse anti-GFAP (SFM248) [DyLight 594], Novus Biologicals (Centennial, CO, USA), CAT: NBP2-34401DL594
ALDH1L1	ALDH1L1 Antibody (2E7) [DyLight 680], Novus Biologicals (Centennial, CO, USA), CAT: NBP2-50033FR

## Data Availability

All data supporting the findings of this study are available within the paper.

## Abbreviations

Aβ	amyloid-β
aCSF	artificial cerebrospinal fluid
AD	Alzheimer’s disease
ALDH1L1	aldehyde dehydrogenase 1 family member L1
AUC	area under the receiver operating characteristic
AUCCi	95% confidence interval of the AUC
BSA	bovine serum albumin
CD11b	cluster of differentiation 11b
CD45	cluster of differentiation 45
CD163	cluster of differentiation 163
DAM	disease-associated microglia
DMEM	Dulbecco’s Modified Eagle Medium
DMSO	dimethyl sulfoxide
EDTA	ethylenediaminetetraacetic acid
EL	escape latency
F4/80	EMR1 (EGF-like module-containing-mucin-like hormone receptor-like 1)
FACS	fluorescence-activated cell sorting
fEPSP	field excitatory postsynaptic potential
GFAP	glial fibrillary acidic protein
HBSS	Hanks’ Balanced Salt Solution
IL-1β	interleukin-1β
LTP	long-term potentiation
P2RY12	purinergic receptor P2Y12
PBS	phosphate-buffered saline
PFA	paraformaldehyde
TG	transgenic
TMEM119	transmembrane protein 119
TREM-2	triggering receptor expressed on myeloid cells 2
WCM	water cross maze
WT	wildtype
